# Starvation-induced changes in the proteome and transcriptome of the salivary glands of leech (*Hirudo nipponia)*

**DOI:** 10.1371/journal.pone.0304453

**Published:** 2024-06-26

**Authors:** Meixiang Cai, Hongying Shen, Yueting Xing, Weimin Wang, Feng Guan, Yuanyuan Luo

**Affiliations:** 1 College of Life Sciences, China Jiliang University, Hangzhou, Zhejiang, China; 2 School of Medicine, Zhejiang University, Hangzhou, Zhejiang, China; 3 Women’s Hospital School of Medicine, Zhejiang University, Hangzhou, Zhejiang, China; Universidade Federal do Rio de Janeiro, BRAZIL

## Abstract

*Hirudo nipponia* is an important medicinal animal in China. Its salivary gland secretions contain a variety of protein bioactive substances. Investigations of its salivary glands are of great significance in the study of the medicinal value and mechanism of leech secretions. Illumina RNA-Seq technology was used to perform transcriptome sequencing of salivary gland tissue of *H*. *nipponia* under starvation (D30) and fed (D0) states. A total of 2,650 differentially expressed genes (DEGs) were screened. Using the label-free protein quantification technique and bioinformatics analysis, the expression of differentially expressed proteins (DEPs) in the salivary gland tissue of *H*. *nipponia* was compared. A total of 2,021 proteins were identified, among which 181 proteins were differentially expressed between the starvation and fed states, with 72 significantly upregulated and 109 significantly downregulated. The salivary glands of *H*. *nipponia* synthesized protein-based active substances after 30 days of starvation and adapted to the starvation environment by weakening respiratory activity and reducing metabolic activity to reduce energy expenditure. Energy was produced by glycolysis and the tricarboxylic acid cycle for the synthesis of substances such as antibiotics. This study combined transcriptome and proteome sequencing data to provide a data reference for an in-depth study of the regulatory mechanism of salivary gland secretions of *H*. *nipponia* under starvation stress by analyzing DEGs and DEPs.

## Introduction

*H*. *nipponia* is a leech, and it is the only blood-sucking species among the 3 species of leeches included in the Pharmacopoeia of the People’s Republic of China [1]. It belongs to the phylum Annelida, the class Clitellata, the order Arhynchobdellida, the family Hirudinidae, and the genus Hirudo. Leech blood-sucking therapy dates to 1500 BC [2], and the medicinal value of leeches was recorded in China as the early Han Dynasty. *H*. *nipponia* live on the blood of mammals and amphibians. Due to their special eating habits, their salivary glands contain a variety of bioactive components, such as the anticoagulant factor calin [3, 4]; the anti-inflammatory factors bdellin [5] and eglins [6]; proteins with antibacterial activity, e.g., hyaluronidase [7]; platelet aggregation inhibitory molecules [8]; antitumor molecules [9]; the anesthetic active substances nicotine and enkephalin [10]; and hypolipidemic molecules [11–13]. Studies on the active components of blood-sucking leeches are mostly found in foreign species, especially *Hirudo medicinalis*; previous studies have mainly focused on clinical applications [14, 15], the isolation and identification of several bioactive substances and the efficacy and mechanism of processed traditional Chinese medicine [16], for example, hirudin has great potential as a coating for vascular stents to treat thrombi. For *H*. *nipponia*, a predominant blood-sucking leech in China, there are many studies on its biological characteristics and anticoagulant activity, but studies on its salivary glands and salivary proteins are rare. In recent years, the rapid development of high-throughput sequencing technology and proteomics technology has provided new approaches and methods for the study of *Hirudo medicinalis*. Transcriptomics and proteomics can systematically reveal the regulatory patterns of gene expression in cells and the change mechanisms of living organisms in specific states. Liu [17] investigated salivary secretions of *Poecilobdella javanica*, *Whitmania pigra*, and *Haemadipsa cavatuses* using combined transcriptomic and proteomic methods. The data revealed that more than 200 bioactive molecules are involved in the leech sucking pathway. Using joint transcriptome and proteomics analysis of the salivary glands of the leech *Whitmania pigra*, Ren [18] discovered a novel protease-activated receptor 1 (PAR1) inhibitor (pigrin), which selectively inhibits platelet aggregation stimulated by PAR1 agonists and collagen.

Due to the uncertainty of natural conditions in the field, the diet of leeches is often unevenly distributed, which can lead to starvation stress, resulting in a range of physiological responses. The aim of this study was to use transcriptome sequencing technology and label-free proteomics technology to better understand the working mechanism of salivary gland tissue of *H*. *nipponia* in response to starvation stress. The key genes and proteins associated with stimulated secretion and synthesis were screened for the study of the mechanisms of the synthesis and secretion of proteins by the salivary glands of *H*. *nipponia*, with elucidation at the omics level. The results of this study are expected to provide an important theoretical basis for the rearing of *H*. *nipponia* and the extraction of many active substances, such as natural hirudin.

## Materials and methods

### Experimental materials and starvation stress treatments

In this experiment, live *H*. *nipponia* (body mass, 2±0.5 g) were purchased from the Dalian Breeding Farm in Liaoning. After body surface morphological identification, *H*. *nipponia* were reared in the laboratory for 30 days at 26 ± 2°C, and the water was replaced with fresh dechlorinated water daily. A total of 30 healthy *H*. *nipponia* with similar size were selected and randomly divided into two groups: a starvation group (D30) and a fed group (D0). A blood bag was provided to the fed group for feeding until the leeches were full and fell naturally (blood came from edible fresh pig blood, purchased from the market). The salivary gland tissue of 15 leeches from the D30 and D0 groups was dissected to obtain three biological replicates; the samples were snap frozen in liquid nitrogen and stored at -80°C for further analysis.

### RNA extraction, library construction and RNA-sequencing

Total RNA was extracted using the TRIzol method. The cDNA libraries were prepared using the VAHTS Stranded mRNA-seq Library Prep Kit for Illumina V2 (Nanjing Vazyme Biotechnology Co., Ltd., China) and was then paired-end sequenced using the Illumina HiSeq 2500 platform (Illumina, USA) [19]. cDNA library construction and transcriptome sequencing were conducted by Sangon Biotech (Shanghai, China) Co., Ltd.

### Data assembly and gene function annotation

FastQC was used to visually evaluate the quality of the raw data. Trimmomatic was used to process the sequences with connectors and low quality in the raw data to obtain clean data. The clean data were De novo assembled into transcripts using Trinity, according to the De Bruijn Graph (DBG) algorithm with parameters set to min_kmer_cov2 and the rest default. Redundancy was removed using Cd-hit-est (similarity threshold value as default, which was 0.9) [20]. The longest transcript in each transcript cluster was taken as the unigene, which was used as the reference sequence for subsequent analysis. Using BLAST+ in the NCBI database to compare and annotate unigene with NR (NCBI non-redundant protein sequences), Swissprot, TrEMBL databases. Based on the optimal comparison results, the open reading frames (ORF) of unigene were determined according to their priority order, the standard codon table was used to determine their CDS and the encoded amino acid sequence. Meanwhile, the TransDecoder software was used to predict the CDS sequence of unmatched unigene, both of which were used as a subsequent MS matching database. The unigene was used as the reference sequence, Bowtie2 was used to compare the sequencing sequence after quality control with the reference sequence, and the comparison results were calculated by RSeQC.

By calculating the TPM (Transcript Per Million) value to compare the differences between different samples of gene expression. The method also took into account the effect of sequencing depth and gene length, as well as the effect of samples on the reads count. After using TMM to standardize the read count data, DEGseq software was used for differential expression analysis according to expression abundance. Differentially expressed genes (DEGs) were screened using |Fold Change|>2; Q value<0.05 and TPM value ≥ 5 in at least one of the two samples as standards, and GO functional annotation and KEGG signaling pathway analysis were performed for DEGs.

### Protein extraction and enzymatic hydrolysis

The sample was ground into powder after being snap frozen in liquid nitrogen, and then, 1 mL of SDT lysis buffer (4% SDS, 100mM Tris-HCl pH7.6) was added and mixed evenly by pipetting, placed in a boiling water bath for 3 min, and centrifuged at 10,000 × g for 10 min. The supernatant was aspirated for subsequent experiments.

Trypsin digestion was performed using the filter-aided proteome preparation (FASP) method. Ten microliters of protein sample was placed in an ultrafiltration tube, DTT reductant was added to a final concentration of 10 mM, and the samples were shaken at 37°C at 600 rpm for 1.5 h. After the samples cooled to room temperature, iodoacetamide (IAA) was added to a final concentration of 20 mM, and the samples were incubated in the dark for 30 min. Then, the samples were centrifuged at 14,000 × g for 10 min in 100 μL of urea buffe; this process was repeated three times, and the then the samples were washed twice with 100 μL of 25 mM NH_4_HCO_3_ buffer. Finally, trypsin was added (trypsin: protein 1:50), and the samples were incubated at 37°C for 15 h. Then, TFA at a final concentration of 0.5% was added to terminate the enzymatic hydrolysis. The filtrate obtained after ultrafiltration was the protein. The protein was desalted using C18 cartridges. After lyophilization, the protein was redissolved in 40 μL of 0.1% formic acid solution and quantified (OD_280_).

### LC‒MS/MS and bioinformatics analysis

Samples were separated using a Nanoelute HPLC system (Bruker, GER) at a nanoliter flow rate. The mobile phases were solution A (0.1% formic acid, 100% aqueous solution) and solution B (99.9% acetonitrile, 0.1% formic acid) with a linear gradient elution at a flow rate of 300 nL/min. Sample was separated by timsTOF Pro mass spectrometry (Bruker, GER) using a C18 reversed-phase analytical column (Thermo Scientific Easy Column, 25 cm long, 75 μm internal diameter). The mass spectrometer was operated in positive ion mode. The applied electrospray voltage was 1.5 kV, the scan range was m/z 100–1,700, and the data were collected in simultaneous accumulation and continuous fragmentation mode. After mass spectrometry, the original data were merged and retrieved and used for library search identification and quantitative analysis using MaxQuant software (version number 1.6.14).

A database for proteomics research was created using the coding sequences of the salivary gland transcriptome data of *H*.*nipponia* (described above). The search parameters were shown in S1 Table. Differentially expressed proteins (DEPs) were screened using fold change in expression (FC) ≥1.2 or ≤0.833 with P <0.05 as the screening criteria. Cluster3.0 and Java Treeview software were used to perform hierarchical clustering analysis. NCBI BLAST+ software and InterProScan were used to search the protein sequences of the selected DEPs to find homologous sequences, and GO annotation of the sequences were performed. The DEPs were analyzed using the KEGG database and mapped to KEGG pathways.

## Results

### Identification of salivary gland transcriptome and proteome

After sequencing the cDNA from the salivary glands of *H*. *nipponia* and filtering out the low-quality sequences from the raw data, 57,542,906 (D0) and 52,999,370 (D30) clean reads were obtained respectively (S2 Table). Using Trinity to De novo assemble the clean reads into transcripts, a total of 261,051 transcripts were produced. The transcripts ranged in length from 201bp to27,163bp, with an average length of 877.15bp, and an N50 of 1,546 bp. After redundancy removal, a total of 145,981 unigenes were obtained, ranging in length from 201bp to 27,163bp, with an average length of 675.04bp. The N50 of the unigene was 1,127bp (S3 Table). Repeated correlation tests were carried out on the sample (S1 Fig), and three correlation indexes of Pearson, Kendall and Spearman were calculated. The closer the correlation index was to 1, the more similar the sample was. The following section outlines the distinctions among the three: Pearson’s correlation coefficient is used to measure the linear association between two continuously correlated random variables. Both Spearman’s and Kendall’s correlation coefficients are coefficients of rank correlation. The Spearman’s correlation coefficient is used to measure the monotonic association between two variables and is not limited to continuous random variables. The Kendall’s correlation coefficient tests for correlation between two variables from the perspective of whether the two variables are consistent [21].

After proteome sequencing, among all the data, 650,529 secondary spectra were obtained, 49,776 secondary spectra were matched with the database, 9,989 peptides were matched, including 9,607 unique peptides, and a total of 2,021 proteins were identified (S2 Fig). Most of these proteins were hypothetical proteins highly similar to genomic sequences for which predicted coding sequences have not been functionally annotated.

### Screening of DEGs and DEPs

**Fig 1:**
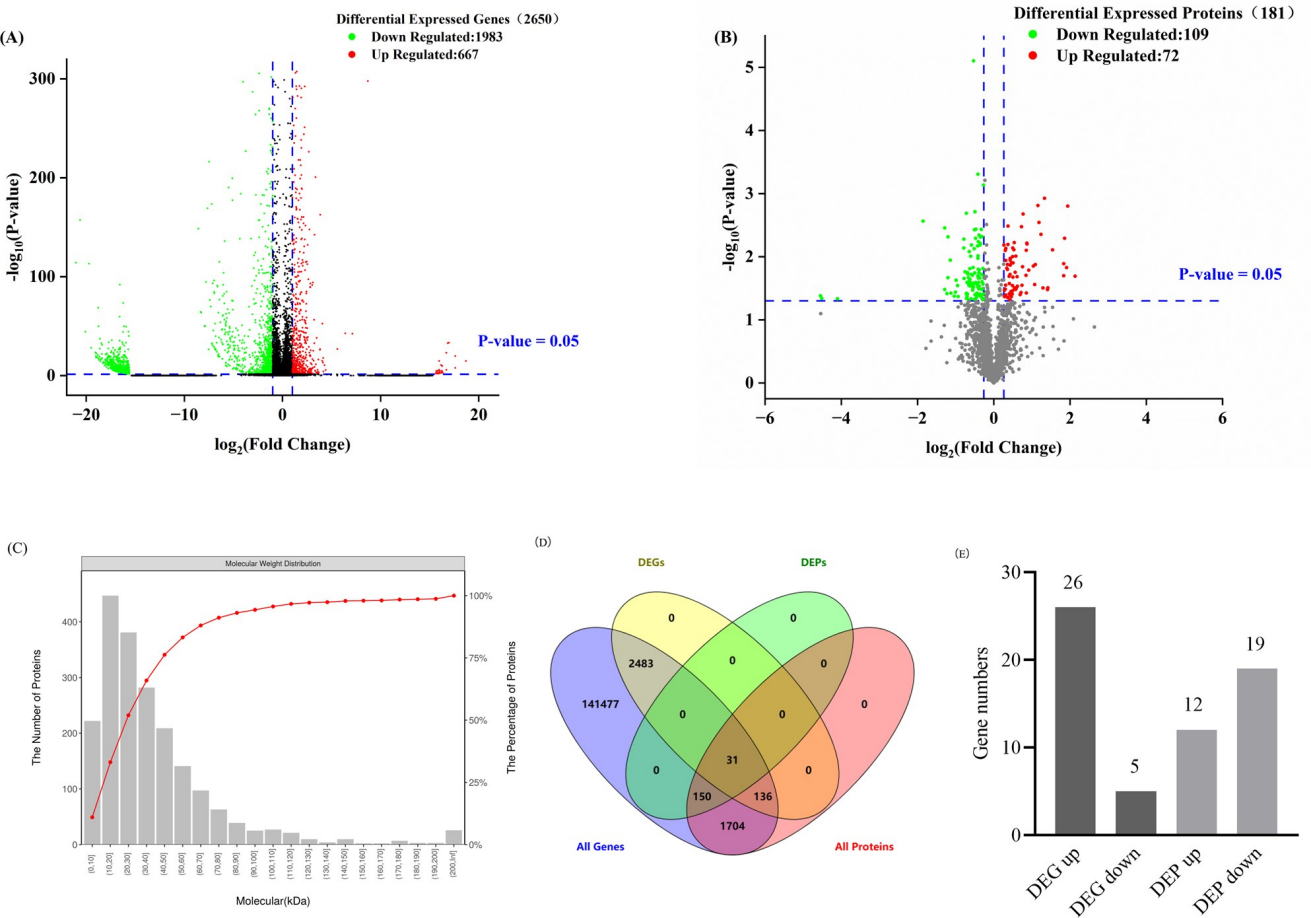
DEGs and DEPs combined analysis. **(**A): Volcano diagram of the distribution of DEGs; (B): Volcano diagram of the distribution of DEPs; (C): Distribution diagram of the relative molecular mass of the identified proteins; (D): Venn diagram of the joint analysis of the transcriptome and proteome; (E): Numbers of upregulated and downregulated genes and proteins by combined analysis of the transcriptome and proteome.

After transcriptome sequencing, a total of 2,650 DEGs were screened, of which 667 genes were significantly upregulated and 1,983 genes were significantly downregulated (Fig 1A). A total of 181 DEPs were identified, with 72 proteins upregulated and 109 downregulated (Fig 1B). In the proteome, approximately 75% of the proteins had a molecular weight of less than 50 kDa (Fig 1C), the distribution of the bands was generally consistent with the SDS-PAGE of the samples (S3 Fig). Of these, low molecular weight proteins (less than 10 kDa) accounted for approximately 11% and 10–20 kDa proteins accounted for the highest percentage of proteins at approximately 22%. The expression of genes and proteins was mainly downregulated. The intersection of DEGs in the transcriptome with DEPs in the proteome is shown in Fig 1D. The results indicated that there were 31 intersecting genes, accounting for 17.13% of the total number of DEPs. Among these genes and proteins, 26 genes and 12 proteins were upregulated, and 5 genes and 19 proteins were downregulated (Fig 1E). All the upregulation and downregulation mentioned above represent D30/D0. Cluster analysis of DEPs (S4 Fig) revealed a strong correlation between the biological replicates in this experiment.

### GO functional annotation and enrichment of DEGs and DEPs

**Fig 2:**
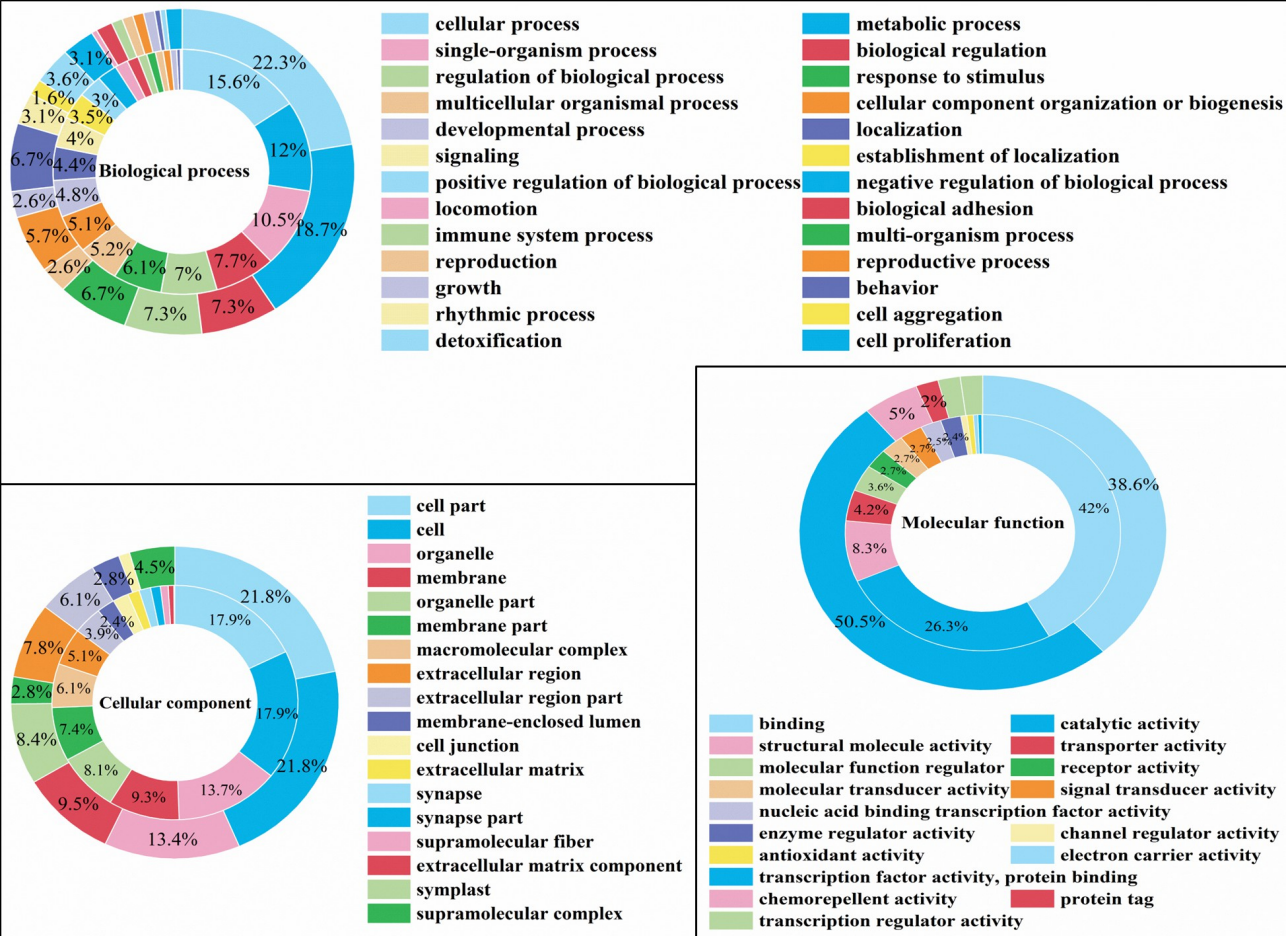
GO functional annotation of DEGs and DEPs. The inner ring is DEPs and the outer ring is DEGs.

GO functional annotation and enrichment analysis were performed on the DEGs and DEPs of the salivary glands of *H*. *nipponia* under starvation stress. The results are shown in Fig 2. In terms of biological processes (BP), 27 DEGs and 22 DEPs were annotated and were mainly involved in cellular processes, metabolic processes, biological regulation, biological process regulation, and stimulation response. In terms of molecular function (MF), DEGs were annotated to 22 entries, and DEPs were annotated to 4 entries, mainly related to catalytic activity, binding, and transportation activities. In terms of cellular components (CC), DEGs were annotated to 22 entries, and DEPs were annotated to 11 entries, mainly from cellular components, cells, organelles, and protein complexes.

GO third- and fourth-level functional analysis of DEPs revealed that DEPs were mainly located in intracellular organelles and the cytoplasm, exerted functions such as protein binding, small molecule binding, organic ring compound binding, ATP binding, and hydrolase activity, and participated in processes such as biosynthesis and cellular metabolism.

**Fig 3:**
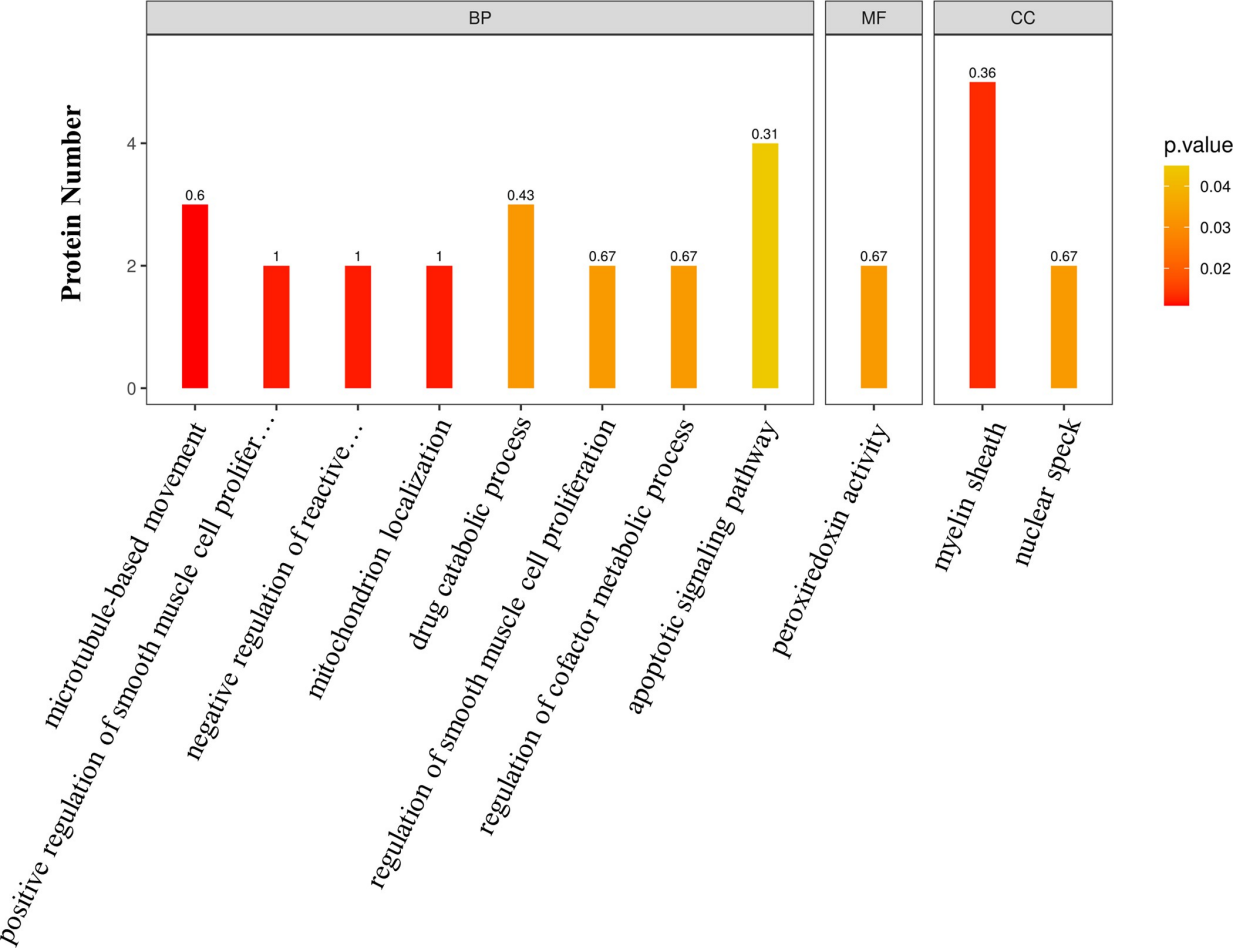
GO functional enrichment of DEPs. The numbers above the bar chart represent the rich factor.

GO functional enrichment of DEPs resulted in a total of 11 enriched terms (Fig 3). In terms of biological processes, the highest levels of enrichment were negative regulation of reactive oxygen species metabolic process and mitochondrial localization. These results suggest that under starvation stress, the mitochondria of *H*. *nipponia* salivary glands limit the excessive production of reactive oxygen species through regulation, thereby reducing the damage to salivary gland tissue caused by oxidative stress.

### KEGG pathway annotation and enrichment analysis of DEGs and DEPs

**Fig 4:**
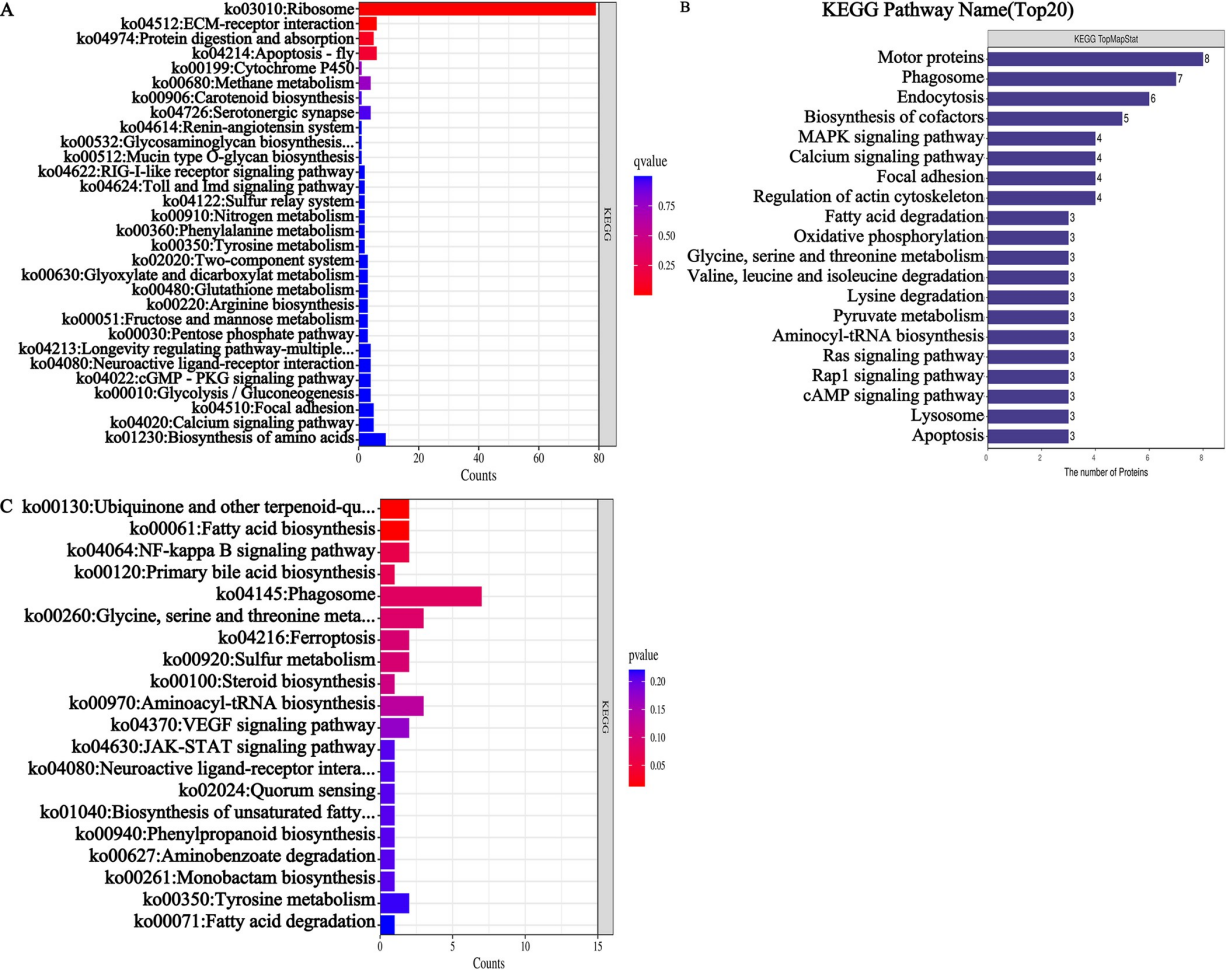
KEGG annotation and enrichment. **(**A): KEGG enrichment pathway of DEGs in *H*. *nipponia* salivary glands under starvation stress; (B): KEGG pathway annotation of DEPs; (C): KEGG pathway enrichment analysis of DEPs.

As shown in Fig 4A, the KEGG pathway enrichment results for the DEGs showed that there were only two significantly enriched pathways, namely, ko03010 (Ribosome) and ko04512 (ECM-receptor interaction), and expression levels were downregulated. The enrichment level of the ribosome pathway was highest. A total of 79 DEGs were annotated in this pathway, and 55 of the 77 DEGs that were downregulated were annotated as ribosomal proteins. The ECM-receptor interaction pathway was enriched for 6 DEGs, all of which were downregulated.

DEPs were annotated to a total of 111 KEGG pathways, of which the top 20 pathways are shown in Fig 4B, with ko04814 (motor proteins), ko04145 (phagosomes), ko04144 (endocytosis), ko00071 (fatty acid degradation), ko00970 (aminoacyl-tRNA biosynthesis), and ko04142 (lysosomes) being the main pathways involved. KEGG enrichment was performed on DEPs (Fig 4C). The significantly enriched pathways were ko00061 (fatty acid biosynthesis) and ko00130 (ubiquitin and other terpenoid-quinone biosynthesis), both of which were downregulated.

## Discussion

Starvation stress is accompanied by a large number of complex physiological processes [22, 23]. Transcriptomics can be used to study the overall structure and function of genes and can reveal the molecular mechanisms of biological processes; proteomics can be used to study the overall changes in protein expression during this process.

Through transcriptomics, we found significant DEGs related to the expression of ribosomal proteins. Ribosomal proteins are the most important components of ribosomes and play important roles in ribosomal translation and protein synthesis. Heeseon et al. [24] found that cells significantly inhibit the translation of ribosomal proteins under acute starvation stress. After feeding, the salivary glands of *H*. *nipponia* synthesize a large amount of protein bioactive substances; these substances are stored for the next feeding and used for storing sucked blood in the body. This indicates that after 30 days of starvation, *H*. *nipponia* salivary glands synthesize secretory proteins and reduce biosynthesis to reduce energy expenditure. Shi et al. [25] used fluorescence quantitative PCR to study the expression level of hirudin in the salivary glands of *H*. *nipponia* during different feeding stages. The results showed that the hirudin content peaked after the second day of food ingestion and then decreased with prolonged fasting time.

The extracellular matrix (ECM) is not only scaffold protein for cell structure but also a special first messenger that senses and transmits extracellular information into cells and is capable of regulating various cellular functions [26]. Starvation stress can reduce signal transduction by inhibiting the ECM-receptor interaction pathway, thereby regulating the progression of cell development.

In this study, proteomics identified that the pathway with the highest degree of enrichment was KEGG ko00061 (fatty acid biosynthesis) and that ko00071 (fatty acid degradation) and ko00970 (aminoacyl-tRNA biosynthesis) were downregulated, results that are consistent with those of other studies, i.e., blood sucking activates fatty acid metabolism and amino acid biosynthesis [17, 27]. Another significantly enriched pathway was ko00130 (Ubiquinone and other terpenoid-quinone biosynthesis), which was downregulated. The reduction in ubiquinone, i.e., coenzyme Q (CoQ), reduces respiratory chain transmission and the energy supply in cells, indicating that the vital activity of *H*. *nipponia* decreases under starvation stress to minimize energy consumption.

According to the KEGG pathway analysis, 8 upregulated DEPs were annotated to ko04814 (motor proteins). Motor proteins are the carriers of intracellular material transport granules and vesicles. We speculate that after being subjected to starvation stress, the salivary gland cells of *H*. *nipponia* began to synthesize a large amount of biologically active proteins. The protein synthesis and transport processes were carried out through vesicles, and dynein hydrolysis of ATP generated energy to drive vesicles along microtubules or the microfilament cytoskeleton, facilitating targeted delivery to subcellular structures for action [28].

Seven upregulated DEPs that were annotated to ko04145 (Phagosome), and 6 upregulated DEPs were annotated to ko04144 (Endocytosis). The main source of nutrition for *H*. *nipponia* is macromolecules in blood. These macromolecules enter salivary gland cells through the endocytosis of phagosomes and are then digested and decomposed into small molecules by ko04142 (lysosome); then, they are used for the synthesis of numerous biologically active substances for the next feeding and for the storage of sucked blood. This indicates that under starvation stress, the absorption and utilization of stored blood improves in *H*. *nipponia*.

Other upregulated pathways included ko00010 (glycolysis), ko00020 (citrate cycle/TCA cycle) and ko00261 (monobactam biosynthesis by monocyclic β-lactam antibiotics). The first two pathways produce a large amount of ATP that can be used to synthesize biologically active substances, and many bioactive substances with anti-inflammatory and antibacterial effects can be synthesized through the monocyclic β-lactam antibiotic pathway.

Using gene annotation, protein quantification and differential analysis, important genes and proteins were screened (Table 1), including several important biologically active proteins, such as hyaluronidase, destabilase, hirudin, bdellin-KL, and bdellin B-3, and their expression levels in the transcriptome and proteome were basically consistent. Bdellin-KL and bdellin B-3 are both atypical Kazal-type inhibitors that can inhibit the effect of trypsin-plasmin [29, 30]. The quantitative results showed that the expression levels of bdellin-KL and bdellin B-3 both decreased after *H*. *nipponia* was subjected to starvation stress, indicating that they can stimulate the synthesis of antifibrotic proteins. Hirudin is a high-efficiency natural thrombin inhibitor extracted from leech salivary glands, and it is also the most studied active ingredient in leeches because of it various pharmacological effects, such as anticoagulant, antithrombotic, antitumor, and antifibrotic effects [31]. Leech hyaluronidase has strong antibacterial properties and can specifically target the β-1,3-glucosidic acid bond in hyaluronic acid [7, 32], thus lysing bacterial and fungal capsules and forming antigen [33, 34]. In addition, leech hyaluronidase is superior to mammalian hyaluronidase in that it can rapidly absorb and diffuse the drug through the skin and is not inhibited by heparin. Destabilase is an endopeptidase with antithrombotic and antibacterial effects [35] that achieves its anticoagulant effect by inhibiting fibrinogen [36]. Additionally, it can also act as a lysozyme to achieve antibacterial effects by digesting the β-1,4-glycosidic bond in peptidoglycan on the bacterial cell wall. These three proteins are all important active components in leech salivary gland secretions, and their expression levels were all upregulated, indicating that starvation stress promoted the synthesis of these anticoagulant, antithrombotic, and antibacterial components.

**Table 1:** Important DEGs and DEPs in salivary gland tissue of *H*. *nipponia*.

Gene ID	Log_2_ (fold change) ^a^	Protein ID	Protein name	Fold change ^b^	Molecular weight/kD
TRINITY_DN69599_c1_g2	1.5970	AHV78514.1	Hyaluronidase	1.6145	24.599
TRINITY_DN58972_c0_g1	1.2597	QDZ37419.1	Hirudin	1.1902	9.859
TRINITY_DN66645_c3_g1	-1.1145	AAF73890.2	Bdellin-KL	0.8066	6.9569
TRINITY_DN66452_c0_g1	-1.0801	P09865.1	Bdellin B-3	0.3676	6.1414
TRINITY_DN50880_c0_g2	Inf	AAA96144.1	Destabilase	1.6542	14.455

^a^ Log2 (fold change) refer to DEGs

^b^ Fold change refer to DGPs.

The quantitative proteomics results were used to screen proteins associated with physiological activities (Table 2). Progranulin (PGRN) is a signal peptide-mediated growth factor secreted by cells, and it participates in various biological processes and disease states [37]. Some studies have found that it can be involved in the development of endoplasmic reticulum (ER) stress [38]. However, there are few studies on invertebrates such as leeches. Granulin precursor isoform B was identified by Vizioli et al. in 2007 by the clonal expression of PGRN in the *Hirudo medicinalis* [39]. The expression of this protein increased, indicating that starvation stress may cause the accumulation of misfolded and unfolded proteins in the ER of *H*. *nipponia* salivary glands.

**Table 2:** Some DEPs quantified in the salivary gland tissue of *H*. *nipponia*.

Protein ID	Protein name	Fold Change	Overlap/%	Peptide number	Number of amino acids	Molecular weight/kD
ABV91207.1	granulin precursor isoform B	2.0536	2.7	1	1089	52.889
BAC82446.1	IB hemoglobin, chain IIB	0.6489	52.7	10	160	18.298
BAC82448.1	hemoglobin, chain C	0.7525	28.2	4	168	19.906
BAC82445.1	hemoglobin, chain IIA	0.6574	5.6	1	160	17.798
AAQ81290.1	voltage-gated sodium channel alpha subunit isoform 4, partial	0.7567	3.1	1	319	37.16
AAD29248.1	intermediate filament, gliarin	1.2615	49	22	638	44.527
AAD29246.1	intermediate filament, filarin	1.2897	51.5	19	597	40.384
BAC82449.1	hemoglobin, linker chain 1	0.7354	42.2	3	229	9.4078
BAC84991.1	globin D1 precursor	0.7220	73.1	8	145	16.256
AAB40925.1	neuron-specific protein	3.5826	11.8	1	121	13.737

In this study, a variety of nerve-related proteins were all downregulated, for example, neuron-specific proteins, voltage-gated sodium channel alpha subunit isoform 4, partial, gliarin, and filarin. Gliarin and filarin [40] are two novel intermediate filaments (IFs) in leech neurons. IFs, together with microtubules and microfilaments, constitute most of the cytoskeletal proteins in multicellular organisms. However, in this study, the nerve cord was removed when obtaining samples. Therefore, there are abundant neuronal cells in the salivary glands of *H*. *nipponia* that are used to receive and transmit information to the salivary glands. When leeches feed, the neurons in salivary glands receive and transmit signals to the salivary glands, causing the salivary glands to secrete saliva. In the starvation state, the neurons are in a relatively resting state.

Leech extracellular hemoglobin is composed of three subunits, i.e., a dimer (globin D-1 and globin D-2) and two monomers (globin M-1 and globin M-2) [41], and transports oxygen. Globin D1 precursor is one of its components. Hemoglobin, chain C, hemoglobin, linker chain 1, and hemoglobin, chain IIA were all downregulated, indicating that after the leeches were subjected to starvation stress, oxygen transport decreased, and respiratory activity became slower.

## Conclusions

In this study, the genes and proteins expressed in the salivary glands of *H*. *nipponia* under different feeding conditions were analyzed using transcriptome and proteome sequencing technologies, and relevant genes and proteins were screened. The results showed that after feeding, *H*. *nipponia* reduced its respiratory activity to reduce energy expenditure and generated energy through glycolysis and the TCA cycle to provide energy for the synthesis of monocyclic β-lactam antibiotics. These antibiotics were transported with hyaluronidase, destabilase, hirudin, bdellin-KL, bdellin B-3, and many other biologically active substances through motor proteins. Additionally, changes in physiological responses in multiple parts of the salivary glands of *H*. *nipponia* under starvation stress were also revealed. The results of this study provide an important reference for the cultivation and efficient utilization of *H*. *nipponia* and the active substances of blood-sucking leeches, for studies on the secretion mechanism of salivary glands in *H*. *nipponia*, and for studies of other medical leeches.

## Supporting information

S1 Fig Transcriptome repeat correlation check scatter plot. (TIF)

S2 Fig Proteome identification and quantitative results. (TIF)

S3 Fig Results of SDS-PAGE electrophoresis of proteome samples. (TIF)

S4 Fig Hierarchical cluster diagram of DEPs. (TIF)

S1 Table Maxquant identification and quantitation indexes. (PDF)

S2 Table Statistic summary of sequencing data. (XLSX)

S3 Table Statistic summary of De nove assembly data. (XLSX)
