# Supplementary figures and images for: Starvation-induced changes in the proteome and transcriptome of the salivary glands of leech (Hirudo nipponia)
Source: PLoS One. 2024 Jun 26;19(6):e0304453. doi: 10.1371/journal.pone.0304453 (PMC11207150; doi:10.1371/journal.pone.0304453)

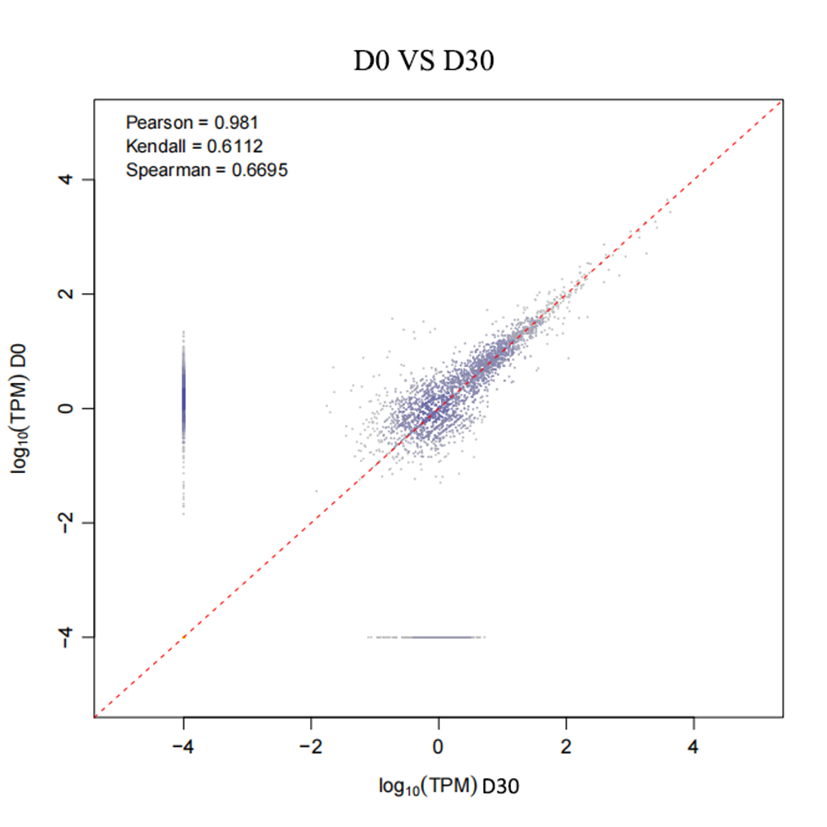

Supplement: S1 Fig — (TIF) [file pone.0304453.s001.tif]

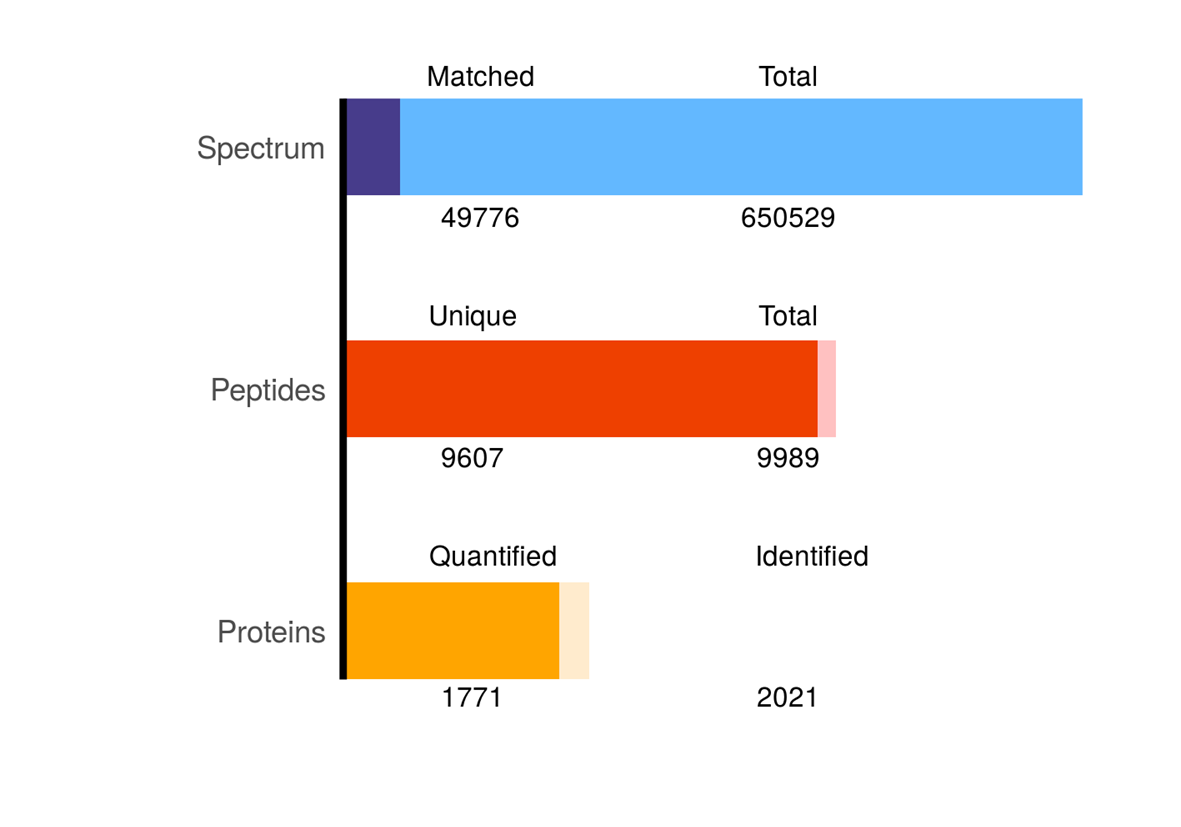

Supplement: S2 Fig — (TIF) [file pone.0304453.s002.tif]

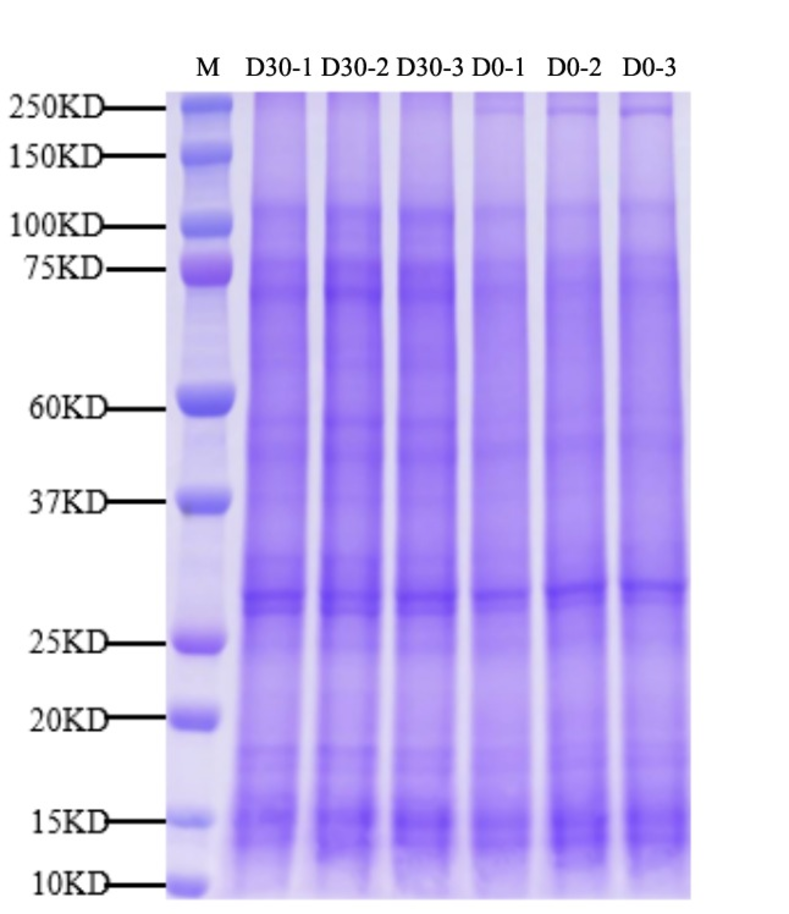

Supplement: S3 Fig — (TIF) [file pone.0304453.s003.tif]

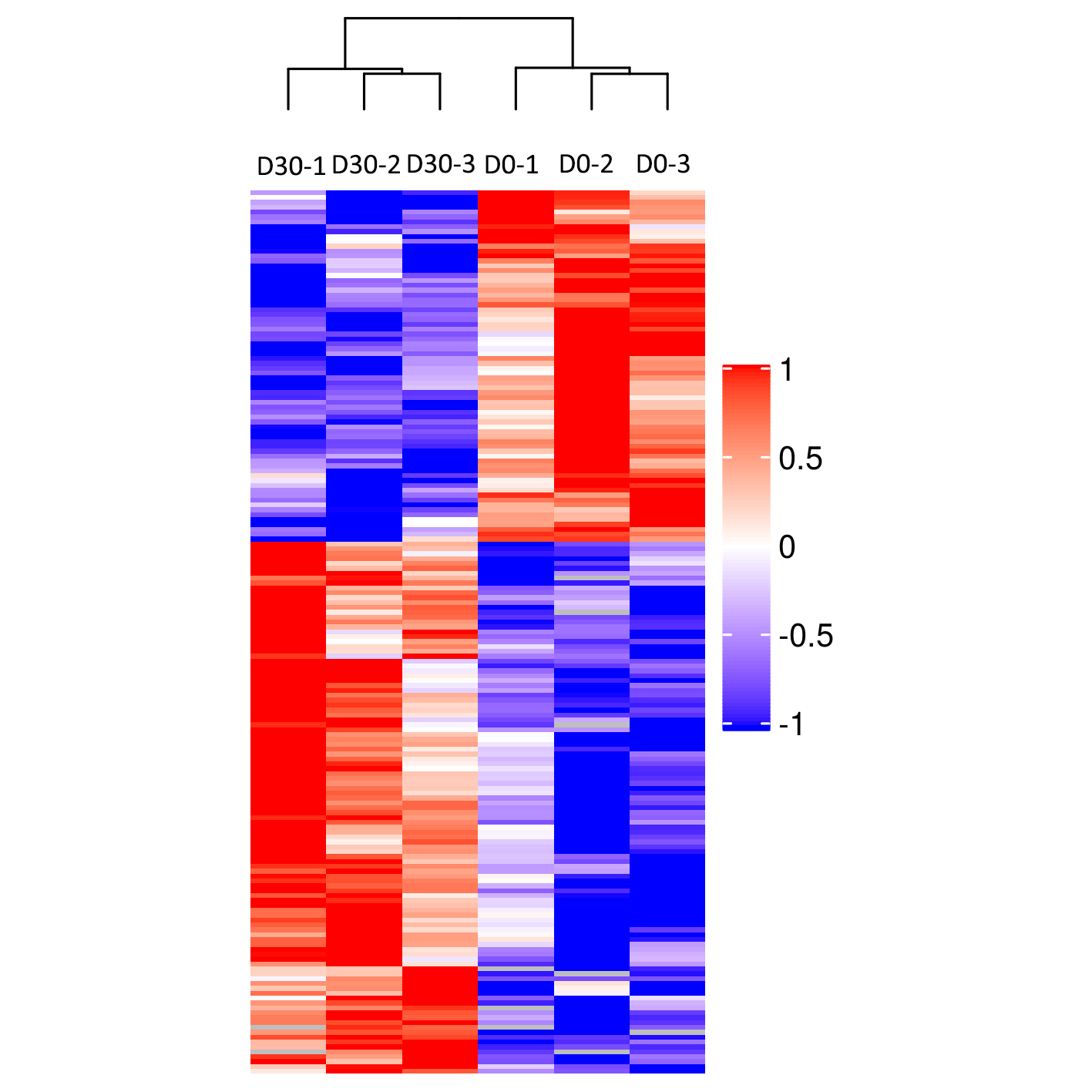

Supplement: S4 Fig — (TIF) [file pone.0304453.s004.tif]
