# Supplementary material for: Starvation-induced changes in the proteome and transcriptome of the salivary glands of leech (Hirudo nipponia)
Source: PLoS One. 2024 Jun 26;19(6):e0304453. doi: 10.1371/journal.pone.0304453 (PMC11207150; doi:10.1371/journal.pone.0304453)
Supplement: S1 Table — (PDF) [file pone.0304453.s005.pdf]

S1 Table Maxquant identification and quantitation indexes

Item	Value
Enzyme	Trypsin
Max Missed Cleavages	2
Fixed modifications	Carbamidomethyl (C)
Variable modifications	Oxidation (M)
Main search	6 ppm
First search	20ppm
MS/MS Tolerance	20ppm
Database pattern	Reverse
Include contaminants	True
protein FDR	≤0.01
Peptide FDR	≤0.01
Peptides used for protein quantification	Use razor and unique peptides
Time window (match between runs)	2min
protein quantification	LFQ
min. ratio count	1
